# Therapeutic Mechanisms of Herbal Medicines Against Insulin Resistance: A Review

**DOI:** 10.3389/fphar.2019.00661

**Published:** 2019-06-14

**Authors:** Jun Li, Litao Bai, Fan Wei, Jing Zhao, Danwei Wang, Yao Xiao, Weitian Yan, Junping Wei

**Affiliations:** ^1^Department of Endocrinology, Guang’anmen Hospital, China Academy of Chinese Medical Sciences, Beijing, China; ^2^Graduate School, Beijing University of Chinese Medicine, Beijing, China

**Keywords:** herbal medicine, insulin resistance, signal transduction, pathway, mechanism

## Abstract

Insulin resistance is a condition in which insulin sensitivity is reduced and the insulin signaling pathway is impaired. Although often expressed as an increase in insulin concentration, the disease is characterized by a decrease in insulin action. This increased workload of the pancreas and the consequent decompensation are not only the main mechanisms for the development of type 2 diabetes (T2D), but also exacerbate the damage of metabolic diseases, including obesity, nonalcoholic fatty liver disease, polycystic ovary syndrome, metabolic syndrome, and others. Many clinical trials have suggested the potential role of herbs in the treatment of insulin resistance, although most of the clinical trials included in this review have certain flaws and bias risks in their methodological design, including the generation of randomization, the concealment of allocation, blinding, and inadequate reporting of sample size estimates. These studies involve not only the single-flavored herbs, but also herbal formulas, extracts, and active ingredients. Numerous of *in vitro* and *in vivo* studies have pointed out that the role of herbal medicine in improving insulin resistance is related to interventions in various aspects of the insulin signaling pathway. The targets involved in these studies include insulin receptor substrate, phosphatidylinositol 3-kinase, glucose transporter, AMP-activated protein kinase, glycogen synthase kinase 3, mitogen-activated protein kinases, c-Jun-N-terminal kinase, nuclear factor-kappaB, protein tyrosine phosphatase 1B, nuclear factor-E2-related factor 2, and peroxisome proliferator-activated receptors. Improved insulin sensitivity upon treatment with herbal medicine provides considerable prospects for treating insulin resistance. This article reviews studies of the target mechanisms of herbal treatments for insulin resistance.

## Introduction

Insulin resistance (IR) is a pathological condition in which target tissues (primarily skeletal muscle, liver, and adipose tissue) have an impaired biological response to insulin stimulation. During IR, the body’s compensatory release of excess insulin to maintain blood sugar stability causes hyperinsulinemia that can progress to type 2 diabetes mellitus (T2D). Prospective studies have highlighted the importance of IR in the pathogenesis of T2D and suggest that IR is the best predictor of future T2D diagnosis (Lillioja et al., 1993). IR and obesity are connected with chronic inflammation in metabolic tissues such as adipose tissue and the liver (Winer et al., 2016). Some studies have pointed out that body mass index is positively associated with IR (Li W. et al., 2014) and inflammation in visceral adipose tissue is a main driver of IR (Lumeng et al., 2007). Closely linked to the epidemic of obesity (Ng et al., 2014), the number of adults with diabetes increased from 108 million in 1980 to 422 million in 2014 (Zhou et al., 2016), and this figure is projected to rise to 642 million people by 2040. There are many vascular and nerve-related complications in diabetes such as diabetes-induced dysregulation of cardiac function, instability of microvasculature of the heart, and increased risk for heart failure (Riehle and Abel, 2016; Levelt et al., 2016; Hinkel et al., 2017). The risk of dementia, Alzheimer’s disease, and cognitive decline are elevated in people with IR (Biessels et al., 2006; Willette et al., 2015; Kullmann et al., 2016) and T2D; the global prevalence of diabetic foot pathologies is 6.3%, and 12.9 to 49.0 million people worldwide have a history of foot ulceration (Armstrong et al., 2017; Zhang P. et al., 2017). These complications bring a tremendous medical and socioeconomic burden. IR is associated with increased risk for other associated disorders, including polycystic ovary syndrome (PCOS), hepatitis C virus, nonalcoholic fatty liver disease (NAFLD), and metabolic syndrome (Diamanti-Kandarakis and Dunaif, 2012; Meex and Watt, 2017; Aytug et al., 2003). Improving IR may provide a therapeutic strategy for controlling T2D, obesity, and many other diseases. Current interventions for IR include intensive lifestyle interventions, thiazolidinedione, DPP-4 inhibitors, and metformin. However, IR is not well controlled and poses a threat to modern society (Kahn et al., 2006). Some herbal medicines such as *Coptis chinensis* Franch (Zhen et al., 2011), *Ganoderma lucidum*, and *Panax ginseng* C. A. Mey result in enhanced insulin sensitivity through modulation of diverse physiological and cellular pathways (Chang et al., 2015; Martel et al., 2017; Bai et al., 2018). For centuries, natural herbs and herbal formulae derived from systemic traditional Chinese medicine theory and practice have been used to treat many kinds of ailments in China. At present, Chinese medicine has received strong support from the World Health Organization and will be included in Chapter 26 of the 11th edition of the Global Medical Program. Chinese medicine also provides treatments for obesity and T2D (D, 2018). In the third century BC, *Huang Di Nei Jing*, the most classic book of Chinese medicine, recorded similar diseases related to diabetes and obesity and provided treatment principles. Now, traditional Chinese medicine is widely used to clinically treat IR. In this review, we explored whether herbs and their formulations or monomers can improve IR and the mechanisms of herbal compounds that increase insulin sensitivity.

## Methodology

According to the Pharmacopoeia of the People’s Republic of China that was revised by the China Food and Drug Administration in 2015, herbal medicine is defined as therapy using herbs and materials derived from botanical herbal products and mineral and animal sources. Interventions that were used in this study include single-flavored herbs and their extracts, active ingredients, and herbal formulas. There were no geographical restrictions on the herbs included.

We reviewed literature (from PubMed) published between July 8, 2013 and July 6, 2018 on IR that had been treated with herbal medicine. The following combination of terms were used as search keywords: “herbal,” “phytochemical,” “phytomedicine,” “natural product,” and “insulin resistance” or “IR.” The search did not exclude articles based on language or status of the publication.

The specified exclusion criteria include: a) case reports, case series, editorials, reviews; b) interventions containing ingredients other than herbs; and c) relevant indicators of IR, such as homeostatic model assessment of IR (HOMA-IR), and IR-index, not involved in the primary and secondary outcomes of clinical trials.

## Results

**Figure 1:**
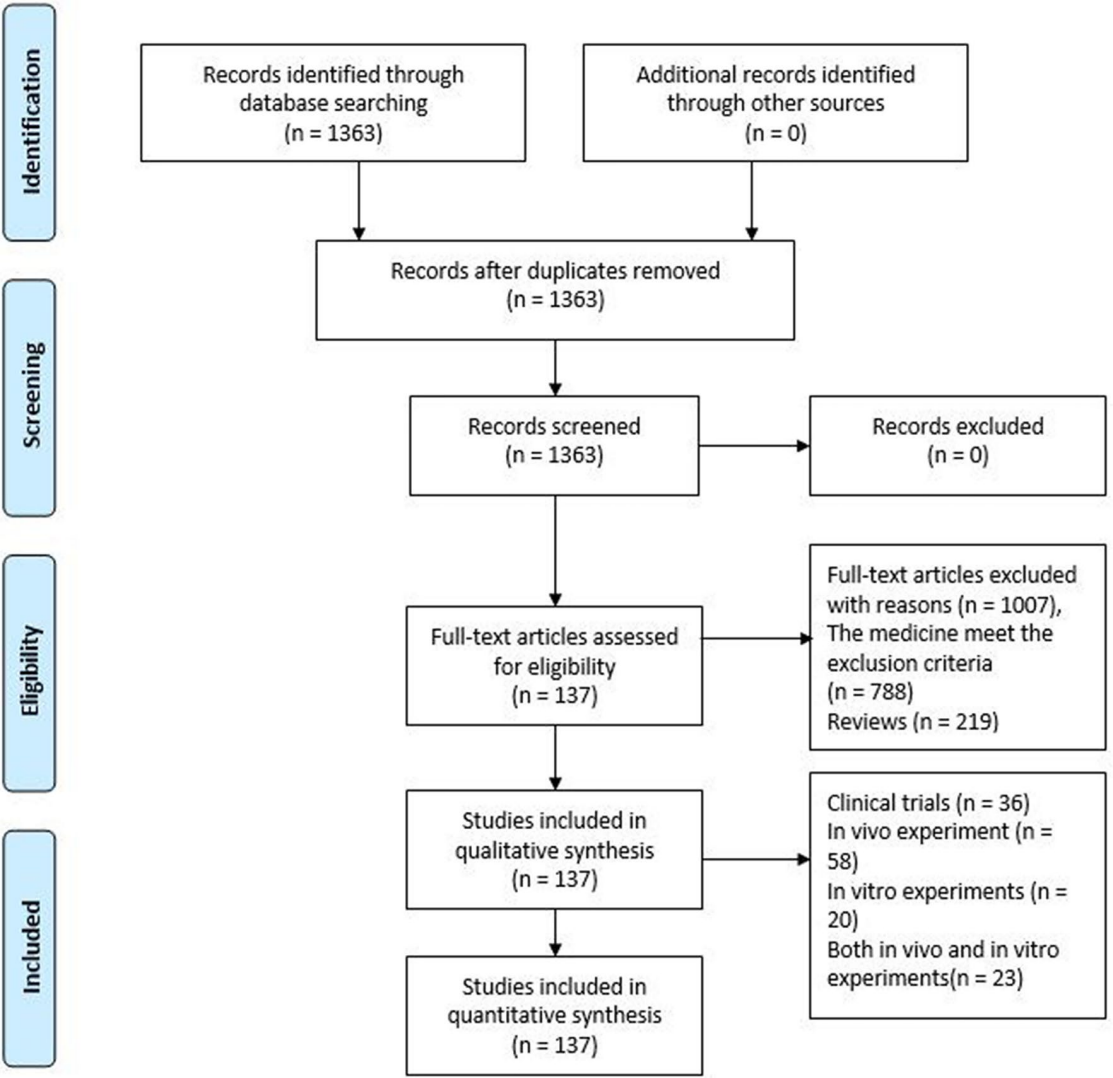
PRISMA 2019 Flow Diagram.

The Preferred Reporting Items for Systematic Reviews and Meta-Analyses (PRISMA) flow chart (**Figure 1**) of article processing shows that our search yielded 1,363 articles, and 1,007 articles were excluded based on the exclusion criteria. After excluding these 1,007 articles, we included 137 articles, including 36 clinical trials, 58 *in vivo* experiments, 20 *in vitro* experiments, and 23 that were a combination of both *in vitro* and *in vivo* experiments. Based on further reading of this literature, we divided 101 *in vivo* and *in vitro* experiments into three parts based on the interventional drug used: active ingredients (31 articles), natural products (38 articles), and herbal formulas (32 articles). The results suggest that most clinical trials (30 articles) indicate that herbal active ingredients, natural products, and herbal formulas, such as JTTZ formula, Jinlida, and Curcumin, have a therapeutic effects on IR. There were a few clinical trials (six articles) that did not support the above results, such as those that used marjoram tea, hydroalcoholic extract of *Juglans regia* (walnut) leaves, *Fraxinus excelsior* L. seeds/fruit extract, garlic extract, bee propolis, red wine polyphenols. On the other hand, we concluded that the effects of herbal medicine on IR may be related to 11 important target molecules that affect insulin signaling, such as insulin receptor substrate, phosphatidylinositol 3-kinase, and glucose transporter.

### Clinical Trials to Improve Insulin Resistance

In clinical trials, participants in the treatment group should be restricted to using herbs alone. If medication other than herbs are being used, then the medication must be administered to both the treatment group and the control group. Participants in the control group should receive hypoglycemic agents, placebo, or life interventions. There is no limit to the number of predetermined herbs, recipes, sample sizes, or duration of treatment.

IR can be accurately assessed by clinical examination. It is generally believed that hyperinsulinemic-euglycemic clamps are the “gold standard” for determining IR, but the complex nature and inherent potential hazards of this technique limit its routine use (Park et al., 2015). Commonly used alternatives are primarily HOMA-IR and quantitative insulin sensitivity check index (QUICKI), which use fasting insulin and glucose concentrations to assess IR and correlate with the results of the clamp study (Matthews et al., 1985). In the 36 clinical trials (**Table 1**) in the above table, most of the drugs examined were herbal formulas, including herbal extracts and active ingredients, and the diseases of concern included T2D, metabolic syndrome, obesity, impaired glucose tolerance, PCOS, and cirrhosis. Most studies have shown that herbal medicine can not only reduce IR but also improve blood sugar, blood lipids, glycosylated hemoglobin, and other biochemical indicators. Indicators of IR in these studies include HOMA-IR, QUICKI, area under the curve of insulin, and IR index. Among them, HOMA-IR was the indicator and was used in 29 studies (29/36, 80%). In order to objectively observe the therapeutic effect of herbs on IR, the changes in IR evaluation indicators before and after treatment in 36 clinical trials are listed in **Table 2**.

**Table 1:** Clinical trials related to insulin resistance.

Compounds/formula	Design	Disease	Dose regimen	Duration	Case/control	Main outcome*	Side effect	Reference
JTTZ Formula	RCT	type 2 diabetic mellitus	0.25g po bid	12 weeks	215/199	HbA1c ↓, T ↓, weight ↓, HOMA-IR ↓	not mentioned	(Yu X. et al., 2018)
Tangyiping Granules (TYP)	RCT	impaired glucose tolerance	10g po bid	3, 12, and 24 months	60/60	NGT ↑, 2hPG ↓, HbA1c ↓, HOMA-IR ↓, TG ↓	not mentioned	(Huang et al., 2016)
Jinlida (JLD)	RCT	impaired glucose tolerance	one bag po tid	12 weeks	32/29	HbA1c ↓, 2hPG ↓, HOMA-IR ↓	no side effect is found	(Shi et al., 2016)
Qingxue Dan (QXD)	RCT	obesity	900mg po qd	8 week	13/13	BMI ↓, TG ↓, WC ↑, total cholesterol ↑, high-density lipoprotein cholesterol ↑, HOMA-IR ↓	not mentioned	(Chung et al., 2016)
Artemisia dracunculus	RCT	impaired glucose tolerance	1000mg po bid	90 days	12/12	HbA1c ↓, AUC of insulin ↓, total insulin secretion ↓, HDL-C ↑, SBP ↓	not mentioned	(Mendez-Del Villar et al., 2016)
Qingre Yangyin Recipe (QRYYR)	RCT	polycystic ovary syndrome	one dose po bid	3 months	30/30	BMI ↓, FINS ↓, 2h INS ↓, HOMA-IR ↓, leptin ↓, LH ↓, PRL ↓, T ↓, APN ↑, FPG ↓, 2 hPG ↓	not mentioned	(Zhang, 2015)
Sancaijiangtang powders	RCT	type 2 diabetic mellitus and vascular dementia	powders po tid	12 weeks	84/84	fasting plasma glucose ↓, fasting insulin ↓, HbA1c ↓, HOMA-IR ↓, plasma nitric oxide ↓, endothelin-1 ↓	not mentioned	(Qiang et al., 2015)
Cinnamon	RCT	type 2 diabetic mellitus	1g po qd	90 days	35/35/35	FPG ↓, 2h PG ↓, HOMA-IR ↓,	not mentioned	(Mirfeizi et al., 2016)
Jinlida	RCT	type 2 diabetic mellitus	9g po tid+ metformin	12 weeks	92/94	HbA1c ↓, FG ↓, 2h PG ↓, HOMA-β ↑, HOMA-IR ↓	not mentioned	(Lian et al., 2015)
Zhenggan Tang decoction	RCT	HBV-induced cirrhotic	125ml po bid	3 months	35/31	leptin ↓, adiponectin ↓, IR-index ↓	not mentioned	(Xu et al., 2015)
Marjoram tea	RCT	polycystic ovary syndrome	1.3–1.5g po bid	1 month	14/11	DHEA-S ↓, fasting insulin levels ↓, HOMA-IR was not reduced significantly	not mentioned	(Haj-Husein et al., 2016)
xin-ju-xiao-gao-fang (XJXGF)	RCT	obesity	170ml po bid	24 weeks	59/55	IR-index ↓, weight ↓	not mentioned	(Zhou et al., 2014)
Yiqi Huaju Recipe (YHR)	RCT	hypertension patients with metabolic syndrome	17.5g po bid	12 weeks	22/21	WC ↓, WHR ↓, 2h PG ↓, FPI ↓, HOMA-IR ↓, average blood pressure amplitude ↑, blood pressure variability ↑, blood pressure load ↑	not mentioned	(Chen et al., 2014)
Ginger (GG)	RCT	type 2 diabetic mellitus	1g po tid	8 weeks	40/41	FBS ↓, HbA1c ↓, HOMA-IR ↓, S% ↑, QUICKI ↑	not mentioned	(Mozaffari-Khosravi et al., 2014)
Curcumin	RCT	type 2 diabetic mellitus	three capsules po bid	6 months	120/120	pulse wave velocity ↓, adiponectin ↑, leptin ↓, HOMA-IR ↓, TG ↓, uric acid ↓, visceral fat ↓, total body fat ↓	no side effect is found	(Chuengsamarn et al., 2014)
Hydroalcoholic extract of *Juglans regia* (walnut) leaves	RCT	type 2 diabetic mellitus	100mg po qd	8 weeks	20/20	weight ↓, body mass index ↓, systolic blood pressure ↓, no significant effect on blood glucose level and HOMA-IR	no side effect is found	(Rabiei et al., 2018)
Yangxin Tongmai Formula (YTF)	RCT	Graves’ disease with impaired glucose tolerance	50ml po tid	60 days	20/20	ISI ↓, HOMA-IR ↓, insulin receptor ↑, plasma insulin levels ↓	not mentioned	(Luo et al., 2018)
Sancai powder	RCT	type 2 diabetic mellitus	200 ml po bid	12 weeks	47/49	HbA1c ↓, FPG ↓, 2hPG ↓, TG ↓, HOMA-β ↓, HOMA-IR ↓, ISI ↓	not mentioned	(Guo et al., 2016)
*Fraxinus excelsior* L. seeds/fruits extract	RCT	non-diabetic overweight/obese	1g po tid	7 weeks	11/6	AUC of insulin ↓, 2hPG ↓, adiponectin: Leptin ratio ↑, fat mass ↓, serum fructosamine ↓, plasma glucagon ↑, no significant changes IR-index and Matsuda Index	no side effect is found	(Zulet et al., 2014)
Berberine	RCT	metabolic syndrome	500mg po tid	3 months	12/12	WC ↓,SBP ↓,TG ↓, AUC of insulin ↓, AUC of glucose ↓, insulinogenic index ↓,Matsuda index ↑	no side effect is found	(Perez-Rubio et al., 2013)
Aloe vera gel complex	RCT	obese prediabetes	1400mg po bid	8 weeks	60/62	weight ↓, HOMA-IR ↓, FBG ↓, serum insulin level ↓	not mentioned	(Choi et al., 2013)
Green tea extract	RCT	type 2 diabetes	500mg po tid	16 weeks	39/38	triglyceride ↓, high density lipoprotein cholesterol ↑, HOMA-IR ↓, Adiponectin ↑, apolipoprotein A1 ↑, apolipoprotein B100 ↑	no side effect is found	(Liu C. et al., 2014)
Cinnamon	RCT	nonalcoholic fatty liver disease	750mg po bid	12 weeks	23/22	HOMA-IR ↓, FBS ↓, total cholesterol ↓, triglyceride ↓, ALT ↓, AST ↓,	not mentioned	(Askari et al., 2014)
Soybean leaf extracts (SLEs)	RCT	prediabetes	2g po qd	12 weeks	15/15	FBG ↓, HbA1c ↓, HOMA-IR ↓	no side effect is found	(Choi et al., 2014)
Nigella sativa	RCT	type 2 diabetes mellitus	2g po qd	12 months	57/57	FBG ↓, HbA1c ↓, HOMA-IR ↓	no side effect is found	(Kaatabi et al., 2015)
Chamomile tea	RCT	type 2 diabetes mellitus	3g po tid	8 weeks	32/32	serum insulin levels ↓, HbA1c ↓, HOMA-IR ↓, total cholesterol ↓, triglyceride ↓	not mentioned	(Rafraf et al., 2015)
DLBS3233	RCT	impaired glucose tolerance	100mg po qd	12 weeks	38/36	HOMA-IR ↓	no side effect is found	(Manaf et al., 2016)
Garlic extract	RCT	type 2 diabetes mellitus	1200mg po qd	4 weeks	13/13	no significant changes in weight, SBP, DBP, total cholesterol, plasma HDL cholesterol, plasma triglycerides, HOMA-IR	indigestion	(Atkin et al., 2016)
Bee propolis	RCT	type 2 diabetes mellitus	300mg po tid	12 weeks	30/27	no significant difference in HOMA-IR	no side effect is found	(Samadi et al., 2017)
Artemisia Extract	RCT	gestational Diabetes Mellitus	400mg po qd	10 weeks	64/65	FPG ↓, serum insulin levels ↓, HOMA-IR ↓	not mentioned	(Sun X. et al., 2016)
Red wine polyphenols	RCT	obesity	300mg po bid	8 weeks	14/15	no significant changes in HOMA-IR, LDL, HDL cholesterol or triglyceride levels	no side effect is found	(Woerdeman et al., 2018)
Fresh yellow onion	RCT	breast cancer	30∼40g po qd	8 weeks	23/23	FPG ↓, serum insulin levels ↓, HOMA-IR ↓	no side effect is found	(Jafarpour-Sadegh et al., 2017)
Gymnema sylvestre	RCT	metabolic syndrome	300mg po bid	12 weeks	12/12	BMI ↓, VLDL ↓, AUC of insulin ↓	no side effect is found	(Zuniga et al., 2017)
Artichoke leaf extract	RCT	metabolic syndrome	1800mg po qd	12 weeks	33/35	no significant in blood pressure, FPG, HOMA-IR ↓	no side effect is found	(Ebrahimi-Mameghani et al., 2018)
Silybum marianum (L). Gaertn. (silymarin) extract	RCT	type 2 diabetes mellitus	140mg po tid	45 days	20/20	FPG ↓, serum insulin ↓, HOMA-IR ↓, triglyceride ↓, HDL-C ↓	no side effect is found	(Ebrahimpour-Koujan et al., 2018)
Green coffee extract	RCT	metabolic syndrome	400mg po bid	8 weeks	22/21	FBS ↓, weight ↓, systolic blood pressure ↓, HOMA-IR ↓	no side effect is found	(Roshan et al., 2018)

AUC, area under the curve; APN, adiponectin; BMI, body mass index; BBT, basal body temperature; DHEA-S, dehydroepiandrosterone-sulphate; FSH, follicle stimulating hormone; FINS, fasting insulin; FPG, fasting blood glucose; FPI, fasting plasma insulin; HDL-C, high-density lipoprotein cholesterol; HOMA-IR, homeostasis model assessment of insulin resistance; HbA1c, fasting glycosylated hemoglobin A1c; ISI, insulin sensitivity index; LH, luteinizing hormone; NGT, normal glucose tolerance; PRL, prolactin; QUICKI, quantitative insulin sensitivity check index; S%, SBP, systolic blood pressure; insulin sensitivity; T, testosterone; TG, triglyceride; WC, waist circumference; WHR, waist to hip ratio; 2h INS, postprandial 2 h insulin; 2h PG, 2 hours plasma glucose; ALT, alanine aminotransferase; AST, aspartate aminotransferase.

**Table 2:** Changes in insulin resistance evaluation indicators before and after treatment in clinical trials.

Compounds/formula	HOMA-IR^1^, AUC of insulin^2^, IRS^3^, QUICKI^4^			Baseline difference
	BT	AT	*p* value**	
JTTZ Formula^1^	T: 1.58 ± 0.72 C: 1.5 ± 0.75	T: 1.39 ± 0.68 C: 1.35 ± 0.67	0.01	NSD
Tangyiping Granules (TYP)^1^	T: 4.02 ± 0.46 C: 3.87± 0.36	T: 3.59 ± 0.31 C: 3.83 ± 0.37	<0.05	NSD
Jinlida (JLD)^1^	T: 2.4(2.0,4.0) C: 2.5(2.1,3.0)	T: 2.41(1.7,3.9) C: 3.0(2.1,3.3)	0.029	NSD
Qingxue Dan (QXD)^1^	T: 164 ± 93 C: 226 ± 160	T: 150 ± 92 C: 205 ± 184	>0.05	NSD
Artemisia dracunculus^2^	T: 56,136 ± 27,426 C: 92,430 ± 55,920	T: 44,472 ± 23,370 C: 94,278 ± 43,230	<0.05	NSD
Qingre Yangyin Recipe (QRYYR)^1^	T: 3.48 ± 2.03 C: 3.81 ± 2.75	T: 2.83 ± 1.52 C: 2.69 ± 1.16	<0.05	NR
Sancaijiangtang powders^1^	T: 6.0 ± 0.8 C: 5.8 ± 1.2	T: 4.6 ± 1.3 C: 3.5 ± 0.8	<0.05	NSD
Cinnamon^1^	T: 8.82 ± 6.59 C: 7.06 ± 5.65	T: 5.58 ± 3.20 C: 5.8 ± 1.2	0.013	NSD
Jinlida^1^	T: 1.32 ± 0.79 C: 1.41 ± 0.79	T: 1.2 ± 0.66 C: 1.31 ± 0.67	0.824	NSD
Zhenggan Tang decoction^3^	T: 1.68 ± 0.21 C: 1.72 ± 0.32	T: 0.92 ± 0.18 C: 1.69 ± 0.44	<0.05	NSD
Marjoram tea^1^	T: 1.53(0.18) C: 1.5(0.25)	T: 1.14(0.14) C: 1.68(0.28)	0.06	NSD
xin-ju-xiao-gao-fang (XJXGF)^1^	T: 8.10 ± 5.32 C: 7.84 ± 5.18	T: 5.48 ± 1.05 C: 9.57 ± 1.45	0.77	NSD
Yiqi Huaju Recipe (YHR)^1^	T: 6.01 ± 4.05 C: 6.50 ± 4.15	T: 4.07 ± 2.80 C: 6.63 ± 4.02	<0.05	NSD
Ginger (GG)^4^	T: 0.316 ± 0.025 C: 0.324 ± 0.031	T: 0.337± 0.303 C: 0.333 ± 0.031	<0.005	NSD
Curcumin^1^	T: 6.12(2-24.1) C: 5.63(1.4-14.9)	T: 5.92(1.4-14.9) C: 2.75(0.9-10.9)	<0.01	NSD
Hydroalcoholic extract of *Juglans regia* (walnut) leaves^1^	T: 3.3 ± 2.7 C: 3.0 ± 1.7	T: 2.9 ± 2.2 C: 2.7 ± 1.4	0.186	NSD
Yangxin Tongmai Formula (YTF)^1^	T: 6.48 ± 1.05 C: 4.26 ± 0.74	T: 4.86 ± 0.54 C: 4.86 ± 0.69	<0.05	NR
Sancai powder^1^	T: 3.2 ± 0.6 C: 3.4 ± 0.6	T: 0.8 ± 0.5 C: 0.7 ± 0.5	<0.05	NSD
*Fraxinus excelsior* L. seeds/fruits extract^1^	T: 5.65(2.68) C: 5.32(3.15)	T: 6.86(5.17) C: 6.05(3.36)	>0.05	NSD
Berberine^2^	T: 92,056 ± 72,148 C: 67,605 ± 18,730	T: 67,407 ± 46,441 C: 86,852 ± 57,863	<0.01	NSD
Aloe vera gel complex^1^	T: 3.4 ± 1.6 C: 3.3 ± 1.2	T: 3.1 ± 0.2 C: 3.5 ± 0.2	<0.01	NSD
Green tea extract^1^	T: 5.4 ± 3.9 C: 5.9 ± 4.5	T: 3.5 ± 2.0 C: 4.7 ± 3.4	0.004	NSD
Cinnamon^1^	T: 2.7 ± 2.0 C: 3.0 ± 1.2	T: 1.7 ± 0.7 C: 3.0 ± 0.0	<0.001	NSD
Soybean leaf extracts (SLEs)^1^	T: 1.08 ± 0.06 C: 1.07 ± 0.08	T: 0.92 ± 0.12 C: 1.18 ± 0.08	<0.05	NSD
Nigella sativa^1^	T: 3.0 ± 0.24 C: 2.5 ± 0.17	T: 2.5 ± 0.18 C: 2.51 ± 0.15	0.004	NSD
Chamomile tea^1^	T: 7.05 ± 2.34 C: 5.24 ± 1.23	T: 4.24 ± 1.95 C: 5.55 ± 1.12	<0.001	NSD
DLBS3233^1^	T: 3.00 ± 1.76 C: 2.76 ± 2.28	T: 2.16 ± 1.17 C: 2.28 ± 1.24	0.001	NSD
Garlic extract^1^	T: 1.89 ± 1.1 C: 2.5 ± 2.0	T: 1.7 ± 0.9 C: 2.0 ± 1.1	0.05	NSD
Bee propolis^4^	T: 0.37 ± 0.03 C: 0.36 ± 0.03	T: 0.34 ± 0.03 C: 0.03 ± 0.33	<0.001	NSD
Artemisia Extract^1^	T: 2.7 ± 1.8 C: 2.8 ± 1.4	T: 1.7 ± 2.4 C: 4.0 ± 1.5	0.031	NSD
Red wine polyphenols^1^	T: 3.2(2.0, 4.5) C: 2.3(1.4, 2.7)	T: 2.9(2.1, 3.8) C: 2.2(1.5, 2.8)	0.72	NSD
Fresh yellow onion^1^	T: 0.052 ± 0.011 C: 0.045 ± 0.01	T: 0.046 ± 0.006 C:0.051 ± 0.01	0.021	NSD
Gymnema sylvestre^2^	T: 61,626 ± 29,700 C: 64,314 ± 34,914	T: 60,468 ± 37,290 C: 90,816 ± 45,336	0.01	NSD
Artichoke leaf extract^1^	T: 3.53(1.44) C: 3.11(1.27)	T: 3.30(1.47) C: 3.63(1.55)	<0.05	NSD
Silybum marianum (L). Gaertn. (silymarin) extract^1^	T: 4.25 ± 2.43 C: 4.49 ± 2.94	T: 2.75 ± 1.19 C: 5.48 ± 3.51	0.008	NSD
Green coffee extract^1^	T: 5.04 ± 3.95 C: 4.71 ± 2.55	T: 3.62 ± 1.83 C: 5.94 ± 5.16	0.024	NSD

AT, after treatment; BT, before treatment; C, control group; NR, no reported; NSD, no significant difference; T, treatment group.

**p value: differences in treatment group before and after treatment. “^1^”, “^2^”, “^3^”, “^4^” indicate that the indicators of insulin resistance in the experiment are “HOMA-IR”, “AUC of insulin”, “IRS” and “QUICKI”.

Two evaluators independently assessed the risk of bias in each study and provided the methodological quality of inclusion in clinical trials according to predetermined criteria in the Cochrane Handbook (**Table 3**). In general, the methodological quality was assessed to be poor. There are few reports of randomized sequence generation and allocation concealment. Fifteen studies (30/36, 83%) detailed how patients were randomized. Only six trials (6/36, 17%) in this study adequately reported the allocation of hidden methods. Twenty-five trials (25/36, 69%) used blinding on their subjects and investigators. Five trials (5/36, 14%) used blinding on their subjects, investigators, and outcome evaluators. The following restrictions should be considered before accepting the conclusion. First, most of the clinical trials included in this review have certain flaws and bias risks in their methodological design, including the generation of randomization, the concealment of allocation, blinding, and inadequate reporting of sample size estimates. Secondly, the duration of 21 trials (21/36, 58%) was greater than or equal to 12 weeks, and 3 trials (3/36, 8%) lasted longer than 6 months. The impact of duration on the results of the study cannot be ignored. Finally, outcome measures for evaluation of major adverse clinical events, such long-term follow-up, were not considered in this review. Therefore, this review indicates that the evidence for both the benefits and harms of herbal treatment for IR is not strong, and it is necessary to rigorously design further trials with high methodological quality to confirm the conclusion.

**Table 3:** Risk of bias of the clinical trials.

Compounds/formula	A	B	C	D	E	F	G	H	Reference
JTTZ Formula	?	–	–	–	+	+	?	?	(Yu X. et al., 2018)
Tangyiping Granules (TYP)	+	+	+	+	?	+	?	?	(Huang et al., 2016)
Jinlida (JLD)	+	–	–	–	?	+	?	?	(Shi et al., 2016)
Qingxue Dan (QXD)	+	?	+	+	–	+	?	?	(Chung et al., 2016)
Artemisia dracunculus	?	–	+	+	–	+	?	?	(Mendez-Del Villar et al., 2016)
Qingre Yangyin Recipe (QRYYR)	–	–	+	–	–	–	?	?	(Zhang, 2015)
Sancaijiangtang powders	+	–	+	–	–	+	?	?	(Qiang et al., 2015)
Cinnamon	+	?	+	+	+	+	?	?	(Mirfeizi et al., 2016)
Jinlida	+	+	+	+	+	+	–	?	(Lian et al., 2015)
Zhenggan Tang decoction	+	–	–	–	–	+	?	?	(Xu et al., 2015)
Marjoram tea	+	–	+	+	+	+	?	?	(Haj-Husein et al., 2016)
xin-ju-xiao-gao-fang (XJXGF)	+	–	+	+	–	+	?	?	(Zhou et al., 2014)
Yiqi Huaju Recipe (YHR)	+	–	+	–	–	+	?	?	(Chen et al., 2014)
Ginger (GG)	+	–	+	+	?	+	?	?	(Mozaffari-Khosravi et al., 2014)
Curcumin	+	+	+	+	–	+	?	?	(Chuengsamarn et al., 2014)
Hydroalcoholic extract of *Juglans regia* (walnut) leaves	+	+	+	+	–	+	?	?	(Rabiei et al., 2018)
Yangxin Tongmai Formula (YTF)^1^	–	–	–	–	–	+	?	?	(Luo et al., 2018)
Sancai powder	+	–	+	–	–	+	?	?	(Guo et al., 2016)
*Fraxinus excelsior* L. seeds/fruits extract	?	+	+	+	+	+	?	?	(Zulet et al., 2014)
Berberine	+	?	+	+	–	+	?	?	(Perez-Rubio et al., 2013)
Aloe vera gel complex	+	–	+	+	–	+	?	?	(Choi et al., 2013)
Green tea extract	+	?	+	+	–	+	?	?	(Liu C. et al., 2014)
Cinnamon	+	–	+	+	–	+	?	?	(Askari et al., 2014)
Soybean leaf extracts (SLEs)	+	–	–	–	–	+	?	?	(Choi et al., 2014)
Nigella sativa	+	–	+	–	–	+	?	+	(Kaatabi et al., 2015)
Chamomile tea	+	–	+	–	–	+	?	?	(Rafraf et al., 2015)
DLBS3233	?	–	+	+	–	+	?	?	(Manaf et al., 2016)
Garlic extract	?	–	+	+	–	+	?	?	(Atkin et al., 2016)
Bee propolis	?	–	+	+	–	+	?	?	(Samadi et al., 2017)
Artemisia Extract	+	–	+	+	–	+	?	?	(Sun X. et al., 2016)
Red wine polyphenols	+	–	+	+	–	+	?	?	(Woerdeman et al., 2018)
Fresh yellow onion	+	–	+	+	+	+	?	?	(Jafarpour-Sadegh et al., 2017)
Gymnema sylvestre	+	–	+	+	–	+	?	?	(Zuniga et al., 2017)
Artichoke leaf extract	+	–	+	+	–	+	?	?	(Ebrahimi-Mameghani et al., 2018)
Silybum marianum (L). Gaertn. (silymarin) extract	+	–	+	+	–	+	?	?	(Ebrahimpour-Koujan et al., 2018)
Green coffee extract	+	+	+	+	?	+	?	?	(Roshan et al., 2018)

A, Adequate sequence generation; B, Concealment of allocation; C, Blinding (patient); D, Blinding (investigator); E, Blinding (assessor); F, Incomplete outcome data addressed (ITT analysis); G, Free of selective reporting; H, Other potential threat to validity; +, Low risk; –, High risk; ?, Unclear.

It must be mentioned that six clinical trials (*Juglans regia* leaves, marjoram tea, *Fraxinus excelsior* L. seeds/fruit extract, garlic extract, bee propolis, and red wine polyphenols) have not confirmed the therapeutic effect of herbs on IR. Possible reasons may include that, firstly, all herbal medicines are not necessarily effective for treating IR while also improving the metabolic index and secondly, these two herbs were not studied using conventional methods of their consuming (they were obtained from extraction using ethanol and soaking in boiling water, respectively). This may have altered the concentration and composition of the drug solution. Despite this, the potential therapeutic effects of herbs on IR are worthy of attention.

### Insulin Signal Transduction

The physiology of insulin involves a complex network of signaling pathways that is activated by the insulin receptor (Samuel and Shulman, 2016). Insulin binding to an insulin receptor on a cell triggers autophosphorylation followed by phosphorylation of intracellular receptor substrates 1 and 2 (IRS-1/IRS-2) (Cheng et al., 2013). Several upstream and downstream key signaling molecules in the insulin signaling pathway have been identified, including the phosphoinositide 3-kinase (PI3K)/Akt pathway that is known to be involved in the translocation of glucose transporter 4 (GLUT-4) from intracellular vesicles to cells and promote glucose uptake to adipose tissue and skeletal muscle, eventually decreasing blood glucose levels. Other related pathways include the mitogen-activated protein kinase (MAPK), adenosine monophosphate-activated protein kinase (AMPK), and stress-activated c-Jun-N-terminal kinase (JNK) pathways (Belwal et al., 2017), among others, and these key players in signal transduction processes are potential targets for drug interventions in IR. IR is characterized by multiple defects, with decreases in receptor concentration and kinase activity, PI3K activity (Anitha et al., 2006), the concentration and phosphorylation of IRS-1 and IRS-2 (Hoehn et al., 2008), and glucose transporter translocation (Bogan, 2012). Previous studies have found that abnormalities in insulin signaling pathways caused by lipid metabolism disorders, inflammatory responses, oxidative stress, endoplasmic reticulum stress, and mitochondrial dysfunction lead to IR (Guilherme et al., 2008; Szendroedi et al., 2011; Gurzov et al., 2014; Park E. et al., 2014; Siwicki et al., 2016). Metabolic disorder and inflammation cause IR and promote leukocytes to secrete proinflammatory cytokines, including IL-6 and tumor necrosis factor-α (TNF-α) (Feve and Bastard, 2009; Wen et al., 2011; Wensveen et al., 2015), which provides a framework to understand how physiological stress, obesity, and diet promote IR. We have placed the experimental research on the treatment of IR by herbal medicine into three categories: active ingredients (**Table 4**), natural product (**Table 5**), and herbal formula (**Table 6**). The active ingredient is a relatively single component, and research has proven to play a major role in the therapeutic effects of herbal medicine. The active ingredients listed in **Table 4** are berberine, ginsenoside, astragaloside, polydatin, baicalin, maslinic acid, paeoniflorin, *Lycium barbarum* polysaccharide, dihydromyricetin, atractylenolide, etc. Natural products are also extracted from herbs, but the ingredients are relatively complex. The herbs involved in the natural products in the table include mulberry leaves, *Coptis chinensis*, litchi seed, red ginseng, and *Gastrodia elata* Blume. There are many herbs involved in herbal formulas. Some of these herbs have been used frequently, such as *C. chinensis*, mulberry leaves, *Pueraria montana* lobata, *Salvia miltiorrhiza*, and *Astragalus membranaceus*. Therapeutic targets for these herbs include: insulin receptor substrate, phosphatidylinositol 3-kinase, glucose transporter, AMP-activated protein kinase (AMPK), glycogen synthase kinase 3, MAPKs, JNK, nuclear factor-kappaB (NF-κB), protein tyrosine phosphatase 1B, nuclear factor-E2-related factor 2, and peroxisome proliferator-activated receptors. The results suggest that herbal interventions for IR are mostly multi-targeted, sometimes interfering with the same target through different pathways. Insulin receptor substrate signals transduction. 

**Table 4:** Active ingredients for improving insulin resistance.

Type	Model	Monomer	Inducer	Animal/cell	Major findings	References
*In vivo* and *in vitro*	Insulin resistance	Baicalin	Diet-induced	Mice and 3T3-L1 cell	p38 MAPK, Akt, GLUT4	(Fang et al., 2018)
*In vivo*	Diabetic myocardial hypertrophy	Polydatin	STZ	Mice	NF-κB, PPARβ	(Huang et al., 2015)
*In vivo* and *in vitro*	Diabetic and insulin resistance	Polydatin	High-fat and -sugar diet and streptozocin, palmitic acid	Rat and HepG2 cell	Akt, GSK-3β, IRS	(Hao et al., 2014)
*In vivo* and *in vitro*	Insulin resistance	*Lycium barbarum* polysaccharide (LBP)	High-fat diet	HepG2 cells and C57BL/6J mice	PI3K/Akt, Nrf2, GSK3β, JNK	(Yang Y. et al., 2014)
*In vivo* and *in vitro*	Adiposity and insulin resistance	Maslinic acid (MA)	High-fat diet	C57BL/6J mice and HepG2 cells	Akt, GSK3β	(Liu J. et al., 2014)
*In vivo*	Insulin resistance	LBP-4a	–	OLETF rats	PI3K, p38 MAPK, GLUT4	(Zhao R. et al., 2014)
*In vitro*	Insulin resistance	Paeoniflorin	–	3T3-L1 adipocytes	IRS-1, Akt	(Kong et al., 2013)
*In vivo*	Obesity	Berberine	High-fat diet	Rats	IRS-1	(Liu D. et al., 2018)
*In vivo*	Obesity	Berberine	High-fat diet	Mice	AMPK	(Wang L. et al., 2018)
*In vivo*	Natural aging	Berberine	–	Rats	p-AMPK	(Yu Y. et al., 2018)
*In vitro*	Insulin resistance	Astragaloside IV	Glucose + insulin	HepG2 cells	AMPK	(Wang C. et al., 2018)
*In vitro*	Insulin resistance	Astragaloside IV	Palmitate	C2C12 myotubes	IRS1, Akt	(Zhu et al., 2016)
*In vivo* and *in vitro*	Obesity/insulin resistance	Ginsenoside Rb2	High-fat diet/TNF-α	Mice/3T3-L1 adipocytes	IRS-1, PI3K/Akt, MAPK	(Dai et al., 2018)
*In vivo*	Obesity	Ginsenoside Rg3	High-fat diet	C57BL/6 mice/3T3-L1 pre-adipocyte cell	PPAR	(Lee et al., 2017)
*In vivo*	Insulin resistance	Ginsenoside Re	High-fat diet	C57BL/6 mice	JNK	(Kim et al., 2017)
*In vivo*	Obesity	Ginsenoside Rg5	High-fat diet	ICR mice	JNK	(Xiao et al., 2017)
*In vivo*	Insulin resistance	Dihydromyricetin (DMY)	High-fat diet	Male Sprague–Dawley (SD) rats	GLUT1, AMPK	(Le et al., 2016)
*In vitro*	Insulin resistance	Atractylenolide I (AT-I) and atractylenolide II (AT-II)	LY294002	Mouse skeletal muscle, C2C12 cells	GLUT4, AMPK, PI3K/Akt	(Chao et al., 2016)
*In vivo* and *in vitro*	Insulin resistance	Fudan-Yueyang Ganoderma lucidum (FYGL)	–	ob/ob mice, L6 rat skeletal muscle cells	PTP1B, PI3K/Akt, GLUT4, IRS-1,	(Yang et al., 2018)
*In vitro*	Insulin resistance	α-Methyl artoflavanocoumarin (MAFC)	Insulin	HepG2 cells	PTP1B, PI3K/Akt, IRS-1,	(Jung et al., 2017)
*In vitro*	Insulin resistance	Anthocyanins from Purple Corn	DMEM	3T3-L1 Adipocytes	PPARγ, IRS-1, GLUT4, Akt	(Luna-Vital et al., 2017)
*In vitro*	Insulin resistance	Geniposide	RPMI-1640 medium	HepG2 cells	NF-κB, GLUT-4	(Jiang et al., 2017)
*In vivo*	Insulin resistance	Tartary buckwheat flavonoid fraction (TBF)	High fructose	Mice	Nrf2, GLUT-4, IRS-1,	(Hu et al., 2017)
*In vitro* and *in vivo*	Insulin resistance	Pectic bee pollen polysaccharide (RBPP-P)	High glucose and fatty acids/high-fat diet	HepG2 cells/mice	AMPK	(Li et al., 2017)
*In vitro*	Insulin resistance	Fucosterol	–	HepG2 cells	PTP1B	(Jung et al., 2016)
*In vitro* and *in vivo*	Insulin resistance	Mulberry anthocyanin extract (MAE)	High glucose plus palmitic acid	HepG2 cells/db/db mice	PI3K/Akt	(Yan et al., 2016)
*In vitro*	Insulin resistance	Tartary buckwheat flavonoids (TBF)	High glucose	HepG2 cells	MAPK, Nrf2, IRS-1,	(Hu et al., 2016)
*In vivo*	Insulin resistance	Corosolic acid	High-fat diet	C57BL/6 mice	IRS-1, AMPK, Akt	(Yang et al., 2016)
*In vivo*	Insulin resistance	1-Deoxynojirimycin (DNJ)	–	db/db mice	PI3K/Akt, GLUT-4, IRS-1	(Liu Q. et al., 2015)
*In vivo*	Insulin resistance	Rutin	S961	C57BL/6 mice	GLUT4	(Hsu et al., 2014)
*In vitro*	Insulin resistance	Total phenolic fraction of *Anemarrhena asphodeloides*	Macrophage-derived conditioned medium	Adipocytes	AMPK	(Zhao W. et al., 2014)

**Table 5:** Natural product that improves insulin resistance.

Type	Model	Natural product	Inducer	Animal/cell	Major findings	References
*In vitro*	Insulin resistance	Oligonol	SF-MEM	HepG2 cells	PTP1B, IRS-1, PI3K/Akt, NF-κB	(Bhakta et al., 2017)
*In vivo*	Type 2 diabetic mellitus	Sang-Tong-Jian (STJ)	High-fat diet	KKAy mice	PI3K/Akt, GLUT2, GLUT4, IRS1	(Kuai et al., 2016)
*In vitro*	Insulin resistance	Marein	High glucose	HepG2 cells	IRS-1, Akt, GLUT1, GSK-3β, AMPK	(Jiang B. et al., 2016)
*In vivo*	Metabolic syndrome	The fermented Red ginseng and Red ginseng extracts	High-fructose diet	Rats	IRS-1, GlUT4	(Kho et al., 2016)
*In vivo*	Insulin resistance	Mulberry leaf extract and its Formulation	BW and high-energy diet	SD rats	IRS-1, JNK	(Liu et al., 2016)
*In vivo*	Insulin resistance	*Parkinsonia aculeata* (Caesalpineaceae)	High-fat diet	Mice	AMPKα	(Araujo et al., 2016)
*In vivo*	Insulin resistance	The fruit of *Acanthopanax senticosus* (Rupr. et Maxim). Harms	High-fat diet	Mice	AMPK	(Saito et al., 2016)
*In vitro*	Type 2 diabetic mellitus	*Nymphaea nouchali* Burm. f. (Family - Nymphaeaceae)	MDI	3T3-L1 adipocytes	PPARγ, GLUT4	(Parimala et al., 2015)
*In vivo*	Type 2 diabetic mellitus	*Cordyceps militaris* number 1 (CmNo1)	High-fat diet	Mice	IRS-1, Akt, GLUT4	(Yu et al., 2015)
*In vitro*	Insulin resistance e	6α-Hydroxylup-20(29)-en-3-on-28-oic acid (1)	Dexamethasone (DXM)	3T3-L1 adipocytes	PI3K/Akt, GLUT4	(Qin et al., 2015)
*In vivo*	Insulin resistance and hyperlipidemia	Ergostatrien-3β-ol (EK100)	High-fat diet	C57BL/6J mouse	GLUT4, AMPK	(Kuo et al., 2015)
*In vitro*	Insulin resistance	Coptidis Rhizoma extracts (CRE)	Tumor necrosis factor-α	3T3-L1 adipocytes	PPAR-γ, IRS-1	(Yuan et al., 2014)
*In vivo*	Metabolic syndrome	*Gastrodia elata* Blume (EGB)	High-fructose diet	Rats	AMPK	(Kho et al., 2014)
*In vivo*	Adipose dysfunction and insulin resistance	*Cyclocarya paliurus* leaves extracts (CPE)	Mac-CM	Mice	IRS-1, Akt	(Jiang et al., 2014)
*In vivo* and *in vitro*	Diabetic	*Oroxylum indicum* Vent. (OI)	STZ	Rats and 3T3-L1 adipocytes	GLUT4	(Singh and Kakkar, 2013)
*In vivo*	Diabetic	*Citrus sinensis* fruit peel (CSMe)	High-fat diet and STZ	Rats	PPARγ, GLUT4	(Sathiyabama et al., 2018)
*In vivo*	Type 2 diabetic mellitus	*Nardostachys jatamansi* DC extract (NJE)	Pelletized commercial chow diet	C57BL/KsJ-db/db mice	AMPK	(You et al., 2018)
*In vivo* and *in vitro*	Type 2 diabetic mellitus	Catalpol	High-fat diet and STZ/glucosamine administration	C57BL/6J mice/HepG2 cells	PI3K/Akt, AMPK	(Yan et al., 2018)
*In vivo*	Insulin resistance	Ethyl acetate fraction (EAF)	High-fat diet, STZ,nicotinamide	Rats	IRS1, Akt	(Ooi et al., 2018)
*In vivo* and *in vitro*	Obesity	Epigallocatechin-3-Gallate-Rich Green Tea Extract	High-fat diet	Male c57BL/6 mice/HepG2 cells	AMPK	(Bae et al., 2018)
*In vivo*	Type 2 diabetic mellitus	*M. charantia* ethanol extracts (MCE)	High-fat diet, streptozotocin (STZ)	Rats	JNK, GLUT4	(Ma et al., 2017)
*In vivo* and *in vitro*	Insulin resistance	Caffeic acid phenethyl ester (CAPE)	–	Mice and HepG2 Cell	JNK, NF-κB, IRS1	(Nie and Chang, 2017)
*In vivo*	Type 2 diabetic mellitus	Aged garlic extract (AGE)	–	Tsumura Suzuki mice	AMPK	(Miki et al., 2017)
*In vivo* and *in vitro*	Insulin resistance/type 2 diabetic mellitus	Sea buckthorn fruit oil is rich in palmitoleic acid (POA)	–	HepG2 cells/SD rats	PI3K/Akt, GSK-3β	(Gao et al., 2017)
*In vitro*	Insulin resistance	*Opuntia ficus-indica* var. *saboten* (OFS)	DMEM	L6 muscle cells	AMPK, p38 MAPK, GLUT4	(Leem et al., 2016)
*In vivo*	Obesity	*P. grandiflorus* root ethanol extract (PGE)	High-fat diet	C57BL/6J mice	PPARα, PPARγ,	(Kim et al., 2016)
*In vivo*	Obesity	*Parkinsonia aculeata* (HEPa/EtOAc)	High-fat diet	C57BL/6J mice	AMPK	(Araujo et al., 2016)
*In vivo* and *in vitro*	Insulin resistance	Rhizoma Anemarrhenae extract (TFA)	STZ	Mice/3T3-L1 and Hela cells	AMPK	(Han et al., 2015)
*In vivo* and *in vitro*	Insulin resistance	Toona Sinensis leaf (TSL)	High-fat diet/AS160	Mice/C2C12 myotubes	AMPK, PPARγ	(Liu H. et al., 2015)
*In vitro* and *in vivo*	Type 2 diabetes mellitus	Fumosorinone (FU)	–	HepG2 cells/KKAy mice	IRS2, Akt, GSK3β, PTP1B	(Liu Z. et al., 2015)
*In vitro* and *in vivo*	Insulin resistance	Ginseng berry extract (GBD)	DMEM	C57BL/6 mice/C2C12 cell	PPARγ. IRS1, Akt	(Yang et al., 2015)
*In vivo*	Type 2 diabetes mellitus	Bitter melon (BM; Momordica charantia)	High-fat diet	OLETF rats	NF-κB, JNK	(Seo et al., 2015)
*In vivo*	Obesity	*Ginkgo biloba* extract (GbE)	High-fat diet	Rats	IRS1, PTP1B	(Banin et al., 2014)
*In vivo*	Metabolic syndrome	Zingiber officinale	High-fat high-carbohydrate diet	Rats/L6 skeletal muscle cells	AMPK	(Li Y. et al., 2014)
*In vitro* and *in vivo*	Insulin resistance	*M. koenigii* (MK)	High-fat diet	Mice/L6 skeletal muscle cells	GLUT4, Akt	(Pandey et al., 2014)
*In vivo*	Obesity	*Artemisia scoparia* (SCO) and *Artemisia santolinifolia* (SAN)	High-fat diet	C57BL/6J mice	PPARγ	(Richard et al., 2014)
*In vitro*	Insulin resistance	Cinnamon extract (CE)	–	3T3-L1 adipocytes and C2C12 myocytes	AMPK	(Shen et al., 2014)
*In vivo*	Obesity	Extracts of *Artemisia santolinaefolia* (SANT) and *Artemisia scoparia* (SCO)	High-fat diet	C57/B6J mice	AMPK	(Wang et al., 2013)

**Table 6:** Herbal formula that improves insulin resistance.

Type	Model	Herbal formula	Inducer	Animal/cell	Major findings	References
*In vivo*	Type 2 diabetic mellitus	SGY preparation	High-fat diet and STZ	db/db mice	PI3K/Akt, IRS-1, GLUT4	(Xing and Chen, 2018)
*In vivo*	Type 2 diabetic mellitus	Dai-Zong-Fang	−	db/db mice	Akt, IRS-1, AMPK, GLUT4	(Zhu et al., 2018)
*In vitro*	Insulin resistance	Zengye Decoction (ZYD)	Insulin-induced	HepG2 cells	AMPK	(Liu Z. et al., 2018)
*In vivo*	Diabetic	Jia-Wei-Jiao-Tai-Wan (JWJTW)	STZ and a high-sucrose-high-fat diet	Rats	IRS, PI3K, GLUT4	(Chen et al., 2017)
*In vivo*	Insulin resistance and nonalcoholic fatty liver disease	Seyoeum (SYE)	High-fat diet	C57BL/6 mice	IRS-1, IRS-2	(Na et al., 2017)
*In vitro*	Insulin resistance	Wu-Mei-Wan	Palmitate	HepG2 cells	PI3K/Akt, GLUT4, IRS	(Yang et al., 2017)
*In vivo* and *in vitro*	Type 2 diabetic mellitus	Preparation JQ-R	Palmitic acid	KKAy mice and cells	NF-κB, PI3K/AKt, JNK, MAPK	(Liu et al., 2017)
*In vivo*	Type 2 diabetic mellitus	Fenugreek seed and mulberry leaf	High-fat diet and alloxan	Rats	GLUT4	(Kan et al., 2017)
*In vivo* and *in vitro*	Insulin resistance	Erchen Decoction and Linguizhugan Decoction	High-fat diet	Rats	NF-κB, IRS-1	(Zhang H. et al., 2017)
*In vivo*	Chronic partial sleep deprivation, obesity-resistant	Jiao-Tai-Wan (JTW)	High-fat, high-energy diet, environmental noise	SD rats	NF-κB	(Zou et al., 2017)
*In vivo*	Metabolic syndrome	Modified lingguizhugan decoction	High-fat diet	Rat	Akt	(Yao et al., 2017)
*In vivo*	Type 2 diabetic mellitus	Jiang Tang Xiao Ke (JTXK) granule	High-fat diet and STZ	KKAy mice	PI3K/Akt, IRS-1, GLUT4, GSK3β	(Yu et al., 2017)
*In vivo*	Insulin resistance	Jiangzhi Capsule	Liquid fructose	Rats	GLUT4, Akt	(Jiang L. et al., 2016)
*In vivo*	Type 2 diabetes mellitus	Liuwei Dihuang decoction	High-fat diets and STZ	SD rats	PI3K/Akt, IRS2	(Dai et al., 2016)
*In vivo*	Type 2 diabetic mellitus	ZiBu PiYin Recipe (ZBPYR)	High-fat diets and STZ	Rat	GSK3β	(Sun Z. et al., 2016)
*In vivo*	Insulin resistance	Jinlida	High-fat diet	Mice	IRS-1	(Jin et al., 2015)
*In vivo*	Polycystic ovary syndrome	Shouwu Jiangqi Decoction (SJD)	Sodium sulfate prasterone, high-fat diet	SD rats	IRS-1, PI3K	(Wang et al., 2016)
*In vivo*	Pre-diabetic	Tang-Nai-Kang (TNK)		Rats	AMPK, PPARγ	(Li et al., 2015)
*In vivo*	Insulin resistance	Jinlida (JLD)	High-fat diet	Rats	JNK, p38MAPK	(Liu Y. et al., 2015)
*In vitro*	Insulin resistance	Modified Si-Miao-San (mSMS)	Conditioned medium derived from activated macrophages	3T3-L1 adipocytes	NF-κB, AMPK, PI3K, IRS-1	(Yang J. et al., 2014)
*In vivo*	Diabetic atherosclerosis	Gal-geun-dang-gwi-tang (GGDGT)	Western diet	(ApoE-/-) mice	IRS-1	(Lee et al., 2014)
*In vivo* and *in vitro*	Insulin resistance	Gyeongshingangjeehwan 18 (GGEx18)	High-fat diet	C57BL/6J mice and 3T3-L1 adipocytes	AMPK, PPARα	(Oh et al., 2015)
*In vivo* and *in vitro*	Insulin resistance	Salvia-Nelumbinis naturalis (SNN)	HC diet	HepG2 cells and rats	Akt, IRS	(Zhang et al., 2014)
*In vivo*	Insulin resistance	Kangen-karyu and Salviae Miltiorrhizae Radix	−	Rats	PI3K/Akt, p38MAPK, NF-κB	(Park C. et al., 2014)
*In vivo*	Polycystic ovarian syndrome	Bushen Huatan Recipe (BHR)	Dehydroeplandrosterone	Rats	Akt, GSK-3β, GLUT4, IRS-1, PPAR-γ	(Hong and Wu, 2014)
*In vivo* and *in vitro*	Metabolic syndrome	Fu Fang Zhen Zhu Tiao Zhi formula (FTZ)	High insulin and high-fat diet	HepG2 cells and rats	PI3K, IRS-1	(Hu et al., 2014)
*In vivo*	Type 2 diabetic mellitus	Fructus Mume formula and its separated prescription	High-fat diet and STZ	Rats	IRS-1, GlUT-4	(Li et al., 2013)
*In vivo*	Insulin resistance	Refined-JQ (JQ-R)	High-fat diet	C57BL/6J mice	AMPK	(Gao et al., 2014)
*In vivo*	Type 2 diabetes mellitus	TZQ-F	High-fat diet	KKA(y) mice	PPARγ, IRS-1, IRS-2, GLUT1, PI3K	(Nan Xia et al., 2013)
*In vivo*	Type 2 diabetes mellitus	Jiaotai Pill (JTP)	STZ and high fat diet	Rats	PI3K, IRS-1, GLUT4	(Dong et al., 2013)
*In vivo*	Polycystic ovary syndrome	Heqi San	Dehydroepiandrosterone	Female SD rats	PI3K/Akt, GLTU4	(Zhao et al., 2017)
*In vivo*	Type 2 diabetes mellitus	Fructus Mume formula	High-fat diet and STZ	Rats	IRS-1, GLUT4	(Li et al., 2013)

### Insulin Receptor Substrate Signal Transduction

The insulin receptor belongs to the subfamily of receptor tyrosine kinases, including insulin-like growth factor 1 receptors and insulin receptor-related receptors (White, 2003). Most insulin signals promote or regulate phosphorylation of IRS-1 or its homolog IRS-2 *via* tyrosine (Haeusler and Accili, 2008), and IRS-1 is the major substrate of the insulin receptor. IRS mediates insulin action differently in different tissues, with IRS-1 playing a prominent role in skeletal muscle and IRS-2 in the liver (Kido et al., 2000). Studies have shown that liver IRS-1 and IRS-2 have complementary effects in controlling liver metabolism; IRS-1 is more closely related to glucose homeostasis (Bouzakri et al., 2006), and IRS-2 is more closely related to lipid metabolism (Taniguchi et al., 2005). Insufficient expression of IRS-1 and IRS-2 can lead to IR (Tamemoto et al., 1994; Shimomura et al., 2000). Jinlida particles can raise insulin sensitivity in skeletal muscle in fat-induced insulin-resistant ApoE-/- mice by increasing the expression of IRS-1 mRNA and protein (Jin et al., 2015). Treating high insulin-induced HepG2 cells with FTZ *in vitro* upregulated the expression of IRS-1 protein while attenuating *in vitro* glucose levels (Hu et al., 2014). Abnormal phosphorylation of IRS is also an important mechanism of IR.

IRS1 and IRS2 appear to lack intrinsic catalytic activity but contain many serine and tyrosine phosphorylation sites (White, 2003). Serine/threonine phosphorylation of IRS-1 at the phosphorylation site Ser307 may inhibit insulin signaling (Rui et al., 2001) and attenuate tyrosine phosphorylation levels (Saad et al., 1992). Salvia-Nelumbinis naturalis (SNN) improves hepatic insulin sensitivity in rats and increases IRS phosphorylation (Zhang et al., 2014). Tumor necrosis factor-α (TNF-α) reduces insulin receptor substrate tyrosine phosphorylation and is an important mediator of IR in obesity and diabetes (Hotamisligil et al., 1996). Erchen decoction and Linguizhugan decoction reduce the level of TNF-α in diet-induced insulin-resistant rats to improve IR (Zhang H. et al., 2017), similar to the pharmacological action of thiazolidinedione (Peraldi et al., 1997). In addition, degenerative neuropathies such as Alzheimer’s disease (Talbot and Wang, 2014) and multiple system atrophy (Bassil et al., 2017) are also closely related to brain IR caused by blocked IRS signaling. Defects in IRS-1 may cause vascular damage and accelerate the progression of atherosclerosis (Abe et al., 1998), while IRS-2 delays neointimal formation under IR (Kubota et al., 2003). Gal-geun-dang-gwi-tang attenuates endothelial dysfunction by promoting nitric oxide (NO)-cyclic guanosine monophosphate (cGMP) signaling and improves insulin sensitivity in individuals with diabetic atherosclerosis. Gal-geun-dang-gwi-tang was also shown to be associated with restored expression of IRS-1 in the thoracic aorta and skeletal muscle (Lee et al., 2014).

### PI3K/Akt Signaling Pathway Signal Transduction

Glucose is mainly metabolized in insulin-sensitive tissues by two pathways: the classical phosphatidylinositol 3-kinase (PI3K) pathway and the 5’-AMP activating kinase (AMPK) signal transduction pathway (Jeong et al., 2017). Tyrosine phosphorylation of the insulin receptor substrate activates PI3K, and activated PI3K catalyzes 4,5-2 phosphatidylinositol (PIP2) and produces PIP3, which acts as a second messenger that activates Akt (White, 2003). Activated Akt promotes downstream molecules that regulate metabolism. Liuwei Dihaung decoction can be used to treat IR by regulating the PI3K/Akt signaling pathway in the liver of rats with T2D, accompanied by phosphorylation and upregulation of PI3K/Akt pathway-associated proteins (Dai et al., 2016). PI3K is composed of a regulatory subunit p85 and a catalytic subunit p110 (Geering et al., 2007) and has dual activities of phosphatidylinositol kinase and serine/threonine (Ser/Thr) protein kinase. Heterozygous mutations in the PI3Kp85 regulatory subunit gene often result in metabolic disorders such as IR associated with decreased ability to activate PI3K in muscle and adipose tissue (Winnay et al., 2016). Significant damage to PI3K signaling in muscle often results in muscle IR and systemic glucose intolerance (Luo et al., 2006). Central glucagon-like peptide 2 can enhance hepatic insulin sensitivity by activating G3-2R-p85a interactions in PI3K signaling in proopiomelanocortin neurons (Shi et al., 2013). Different proportions of Jiaotai Pill enhance PI3K pathway insulin signaling by upregulating the expression of the PI3K p85 subunit in skeletal muscle, attenuating the development of diabetes in a rat model of T2D (Dong et al., 2013). Akt has three isoforms, of which Akt1 and Akt2 are highly expressed in skeletal muscle and have unique and overlapping functions (Matheny et al., 2018). Overexpression of Akt1 is associated with increased beta cell size and total islet mass (Tuttle et al., 2001), and Akt2 is more important for insulin-stimulated glucose metabolism (Bouzakri et al., 2006). Defects in signaling pathways caused by mutations in the protein kinase *Akt2/PKB gene* often impair the ability of insulin to lower blood glucose in the liver and skeletal muscle (George et al., 2004). Paeoniflorin improves TNF-α-induced IR in adipocytes and is associated with insulin-stimulated Akt phosphorylation recovery in adipocytes (Kong et al., 2013). Akt2 is involved in glucose uptake by insulin-regulated muscle and adipocytes by promoting the transport of GLUT-4 to the cell surface (Ng et al., 2008), as is metformin, which also increases liver Akt phosphorylation and promotes GLUT-4 translocation (Garabadu and Krishnamurthy, 2017). Jiangzhi capsule improved fructose-induced IR and repaired the damaged muscle fiber membrane GLUT-4 cycle by regulating the ratio of phosphorylated Akt to total Akt in the gastrocnemius muscle (Jiang L. et al., 2016). Akt2 is required for hepatic lipid accumulation in obese and insulin-resistant states induced by leptin deficiency or high-fat diet (HFD) (Leavens et al., 2009). Modified lingguizhugan decoction improves liver fat accumulation and IR in rats with metabolic syndrome by inhibiting abnormal increases in leptin and PKB in the liver (Yao et al., 2017).

### Glucose Transporters Signal Transduction

There are currently 13 known sugar transporter proteins (GLUT1-12 and HMIT) encoded in the human genome (Joost and Thorens, 2001). Based on sequence similarity and characteristic elements, the extended GLUT family can be divided into three subfamilies, of which class I contains the glucose transporters GLUT1-4, which all show tissue/cell specific expression (Wood and Trayhurn, 2003). GLUT-4 is highly expressed in adipose tissue and skeletal muscle (Bogan, 2012). The main cellular mechanism for handling exogenous glucose load is insulin-stimulated glucose transport into skeletal muscle, and the primary glucose transporter that mediates this uptake is GLUT-4 (Huang and Czech, 2007). Oxidative stress caused by overnutrition often induces GLUT-4 inactivation by carbonylation and oxidation (Boden et al., 2015), and related gene variants (Stenbit et al., 1997) often lead to GLUT-4 deficiency and downregulation in skeletal muscle and adipose tissue (Zisman et al., 2000; Abel et al., 2001). Exercise and caloric restriction can upregulate GLUT-4 gene expression and increase insulin-induced GLUT-4 transport to the plasma membrane (Richter and Hargreaves 2013; Zanquetta et al., 2003). A novel botanical formula containing standardized extracts of mulberry leaf, fenugreek seed, and American ginseng can attenuate the decrease in GL UT-4 expression induced by an HFD and alloxan (Kan et al., 2017). A key step in the physiological role of GLUT-4 is translocation (Bai et al., 2007), in which GLUT-4 is redistributed from the intracellular pool to the plasma membrane under the regulation of the Akt substrate AS160 rab GTPaseactivating protein (Sano et al., 2007). Studies have shown that cold exposure therapy can significantly increase GLUT-4 translocation in basal skeletal muscle and may be a potential treatment for diabetes (Hanssen et al., 2015). *Oroxylum indicum* stem bark extract significantly enhanced insulin sensitivity in mature 3T3-L1 adipocytes, as evidenced by increased skeletal muscle GLUT-4 translocation (Singh and Kakkar, 2013). GLUT-2 is mainly found in the liver, intestine, kidney, and pancreatic beta cells (Wood and Trayhurn, 2003), and it plays an important role in glucose transport and energy metabolism because it promotes glucose uptake or liver efflux (Thorens et al., 1992). Sang-Tong-Jian, a new formulation of flavonoids and alkaloids from mulberry leaves, improves IR in KKAy mice, which is associated with upregulation of GLUT-2 (liver) gene, and protein expression (Kuai et al., 2016). GLUT-1 is expressed at the highest level in the endothelial tissues of barrier tissues such as blood vessels and blood-brain barriers, and moderate levels of expression are also observed in adipose tissue, muscle, and liver, which play an important role in supplying glucose to organs such as the brain (Deng et al., 2014; Tang et al., 2017). Chinese herbal formula TZQ-F treatment upregulates the expression of related proteins such as GLUT-1, which regulates the potency of insulin action and is beneficial for reducing hyperinsulinemia (Nan Xia et al., 2013).

### AMP-Activated Protein Kinase Signal Transduction

AMPK is a conserved, ubiquitously expressed heterotrimeric serine/threonine protein kinase (Kahn et al., 2005) that plays a key role in regulating cellular energy metabolism (Lage et al., 2008). AMPK integrates nutrient and hormonal signals in peripheral tissues and the hypothalamus and is regulated by multiple hormones such as leptin, adiponectin, ciliary neurotrophic factor, and ghrelin (Minokoshi et al., 2002; Watt et al., 2006; Zhao L. et al., 2015). AMPK plays an important role in regulating food intake, body weight, glucose, and lipid metabolism (Minokoshi et al., 2004). In addition, AMPK can also be activated in response to cellular stress, exercise (Cantó et al., 2010), and drugs (Sasaki et al., 2009). Based on traditional Chinese medicine, Dai-Zong-Fang improves insulin sensitivity in db/db diabetic mice by inhibiting liver lipids and enhancing energy metabolism in skeletal muscle by inhibiting AMPK activation (Zhu et al., 2018). Under physiological conditions, AMPK is mainly present in an inactive form complexed with Mg-ATP, which is more abundant than AMP (Xiao et al., 2011). An increase in AMP concentration activates AMPK, and by phosphorylating the activation loop within the kinase domain (Li et al., 2011), AMPK transitions from an inactive form to a catalytic form: binding of AMP to the c-regulatory domain promotes phosphorylation of the upstream kinase and causes allosteric activation, and inhibition of the dephosphorylation of Thr172 in the kinase domain activation loop regulates AMPK phosphorylation levels (Xiao et al., 2007). Goka fruit supplements improve IR and liver lipid accumulation in mice with HFD-induced obesity by increasing AMPK phosphorylation in the liver. AMPK in adipocytes is critical for maintaining mitochondrial integrity, responding to pharmacological agents and heat stress, and protecting against nutrient overload-induced NAFLD and IR (Kishton et al., 2016; Mottillo et al., 2016). Modified Si-Miao-San positively regulates AMPK phosphorylation to promote basal glucose uptake by 3T3-L1 adipocytes and beneficially improves insulin signaling by inhibiting inflammation in adipocytes (Yang J. et al., 2014). Adiponectin is a major insulin-sensitized adipokine (Kadowaki and Yamauchi, 2011). It has been shown that adiponectin and its receptors AdipoR1 and AdipoR2 enhance glucose and fatty acid metabolism by activating AMPK in peripheral tissues (Kubota et al., 2007). The herbal composition GGEx18 can treat visceral obesity and visceral obesity-related IR by upregulating visceral fat expression of fatty acid oxidation genes. The results show that the expression of fatty acid oxidation genes, including genes encoding adiponectin, AMPK, and others, is significantly increased in mesenteric adipose tissue of 3T3-L1 adipocytes and obese mice (Oh et al., 2015).

### Glycogen Synthase Kinase 3 Signal Transduction

GSK-3 is a ubiquitous cytosolic serine/threonine protein kinase expressed in mammalian tissues as two closely related isoforms: GSK-3α and GSK-3β (Dajani et al., 2001). GSK-3 is constitutively active under resting conditions and regulates human metabolism through phosphorylation of glycogen synthase and other substrates (Kaidanovich and Eldar-Finkelman, 2002). Inhibition of GSK-3 is required for insulin-stimulated glycogen and protein synthesis, and its inhibition is critical for the normal functioning of insulin-activated signaling pathways (Ali et al., 2001). The skeletal muscle GSK-3 activity and its expression level were significantly increased in patients with T2D (Saltiel and Kahn, 2001), and abnormally excessively elevated GSK-3 resulted in further inhibition of glycogen synthase activity. A large body of evidence indicates that GSK-3 inhibitors have therapeutic uses in neurodegenerative diseases, cancer, and T2D (Patel and Woodgett, 2008; Hur and Zhou, 2010; Martinez et al., 2002). Jiangtangxiaoke granules are an effective drug for T2D, since this treatment can regulate the expression of glycogen synthase kinase 3β (GSK3β) by regulating the PI3K/Akt signaling pathway in skeletal muscle of mice with T2D (Yu et al., 2017). Insulin-induced GSK-3 inhibition is mediated through its downstream target protein kinase B (PKB), which phosphorylates and inactivates GSK-3 at Ser9/21 (Cross et al., 1995; Frame et al., 2001). ZiBu PiYin recipe treatment alters insulin signaling in T2DM rats in association with inhibited GSK3β overexpression resulting from increased p-GSK3β levels in the pre-frontal cortex and hippocampus (Sun Z. et al., 2016). Abnormal overactivity of GSK-3 may also limit IR-mediated signaling through phosphorylation of IRS-1. Polydatin significantly increased phosphorylated GSK-3β and increased protein levels of phosphorylated IRS in liver and insulin-resistant HepG2 cells of diabetic rats (Hao et al., 2014). Therefore, GSK-3 inhibitors can be a promising new drug for diabetic IR.

### P38 Mitogen-Activated Protein Kinase Signal Transduction

MAPKs and their downstream targets are important signaling modules for cellular responses to changes in the physical and chemical properties of the environment (Cuenda et al., 2017). It is known that MAPK has at least four subfamilies in mammalian cells: p38 kinase (p38α, β, γ, and δ), extracellular signal-regulated kinase (ERK1/2), ERK5, and Jun amino terminal kinase (JNK1-3) (Gehart et al., 2010). Insulin activates the PI3K/Akt pathway, which is responsible for glucose uptake, and the MAPK pathway, which is critical for IR (Saltiel and Kahn, 2001). MAPK is involved in a variety of processes that control hepatic metabolism (Lawan and Bennett, 2017). Obesity and inflammation-related stress responses in insulin-responsive tissues activate liver MAPKs, which are thought to impair insulin action and lipid metabolism (Hotamisligil and Davis, 2016). MAPK phosphatases (MKPs) can dephosphorylate MAPK to catalyze the inactivation of MAPK (Gehart et al., 2010). Baicalin plays an important role in reversing HFD-induced glucose intolerance and IR in diet-induced obese mice, and its mechanism is associated with downregulation of p-p38 MAPK levels (Fang et al., 2018). Reactive oxygen species (ROS)-mediated activation of p38MAPK stress response signaling has been recognized as one of the causes of insulin signaling damage and hepatic IR (Al-Lahham et al., 2016). Jinlida, a compound preparation based on traditional Chinese medicine, can attenuate oxidative stress and reduce phosphorylation of p38MAPK and JNK in high-fat fed rats, showing antioxidant effects and upregulation of insulin signaling (Liu Y. et al., 2015).

### C-Jun-N-Terminal Kinase Signal Transduction

The JNK is a member of the MAPK family, and three JNK isoforms exist in mammals: JNK1, JNK2, and JNK3 (Lawan and Bennett, 2017). JNK1 and JNK2 are expressed in almost all celRls, including liver parenchymal cells, while JNK3 is mainly expressed in the brain, heart, and testis (Seki et al., 2012). Cytokines, ROS, endoplasmic reticulum stress, and free fatty acids activate JNK (Ozcan et al., 2004; Holzer et al., 2011; Zhao H. et al., 2015) and play a key role in metabolic disorders such as obesity, IR, and T2D (Vallerie and Hotamisligil, 2010). *Lycium barbarum* polysaccharide treatment effectively inhibits phospho-JNK levels in HFD-fed mice and reduces ROS levels *via* the PI3K/AKT/Nrf2 axis, acting as a novel anti-hyperlipide-induced IR oxidizer (Yang Y. et al., 2014). Stress factors such as non-esterified fatty acids are generally thought to induce inhibitory serine phosphorylation of IRS-1 through the JNK pathway and impair insulin signaling (Hirosumi et al., 2002; Gao et al., 2018). Studies have also confirmed that hepatocyte-specific deletion of JNK1 enhances IR (Sabio et al., 2009). Therefore, JNK may positively regulate hepatic insulin signaling, and in other insulin-sensitive organs, JNK negatively regulates insulin action, especially under stress conditions. This is the potential protective effect of mulberry leaf extract (MLE) and a formula consisting of MLE, fenugreek seed extract, and cinnamon cassia extract (MLEF) on hyperglycemia induced by high-energy diet and toxic chemicals in rats and recovery of insulin sensitivity, the most likely mechanism is the upregulation of phosphorylation of JNK and other related proteins in the liver to promote IRS-1 phosphorylation (Liu et al., 2016).

### Nuclear Factor-kappaB Signal Transduction

NF-κB is a sequence-specific transcription factor that is a major regulator of inflammatory responses, including responses to inflammation and oxidative stress (Chiang et al., 2009). In the quiescent state, NF-κB binds to the inhibitor subunit IκB in an inactive form in the cytoplasm. IKK-β is required for activation during acute inflammation. Phosphorylation-activated IKKβ induces phosphorylation of IκB kinase α (Arkan et al., 2005) and IκBα phosphorylates IκB and leads to proteolysis of IκB, which exposes nuclear recognition sites for NF-κB. NF-κB is translocated into the nucleus, resulting in the expression of related target genes such as inflammatory cytokines (Cai et al., 2005). Thus, blocking NF-κB signaling improves IR and prevents the development of diabetes (Wang et al., 2014). Jiao-Tai-Wan, composed of Rhizome Coptidis and Cortex Cinnamomi, reversibly increases markers of systemic inflammation and IR caused by sleep loss in Sprague-Dawley rats, and these changes are related to downregulation of NF-κB mRNA expression in peripheral blood mononuclear cells (Zou et al., 2017). NF-κB may represent an attractive therapeutic target for obesity, IR, diabetes, and other complications associated with these diseases.

### Protein Tyrosine Phosphatase 1B Signal Transduction

Protein tyrosine phosphatase 1B belongs to the protein tyrosine phosphatase (PTP) family (Tiganis, 2013) and has a catalytic domain characterized by an 11 amino acid sequence motif containing cysteine (Cys215) and arginine (Arg221) (Haque et al., 2011). These residues are critical to the catalytic activity of the enzyme. As a negative regulator of the insulin signaling cascade, PTP1B overexpression inhibits tyrosine phosphorylation of IR and IRS-1, enhances serine phosphorylation, thereby terminating insulin signaling (Johnson et al., 2002). Oligonol, a low molecular weight polyphenol mixture derived from lychee fruit, can significantly reduce PTP1B expression and reduce serine phosphorylation of IRS-1, improving insulin sensitivity in insulin-resistant HepG2 cells (Bhakta et al., 2017). In general, PTP1B inhibitors are a promising class of insulin sensitizers.

### Nuclear Factor-E2-Related Factor 2 Signal Transduction

Nuclear factor erythrocyte 2-related factor 2 (Nrf2) is a key regulator of antioxidant signaling and plays a crucial role in maintaining redox homeostasis (Seo and Lee, 2013). Under physiological conditions, Nrf2 remains in the cytoplasm by binding to the endogenous inhibitor Keap1, which mediates rapid activation of the proteasome and subsequent degradation of Nrf2 (Zhang et al., 2015). Under exogenous and endogenous oxidative stress, Nrf2 becomes stable and released from the Keap1/Nrf2 complex, Nrf2 degradation is inhibited, and Nrf2 accumulates in the nucleus, synergistically enhancing the expression of various genes encoding antioxidant enzymes (Bhakkiyalakshmi et al., 2015). Many studies have shown that increased Nrf2 signaling can inhibit oxidative stress and improve insulin and leptin resistance (Yagishita et al., 2017). *L. barbarum* polysaccharide, an antioxidant from wolfberry, increases Nrf2 phosphorylation in livers of HFD-fed mice and HepG2 cells by inducing PI3K/AKT signaling and induces Nrf2/ARE signaling to reduce oxidative stress and maintain peripheral insulin sensitivity (Yang Y. et al., 2014).

### Peroxisome Proliferator-Activated Receptor Signal Transduction

Peroxisome proliferator-activated receptors (PPARs) are nuclear receptors involved in the transcriptional control of genes encoding proteins involved in adipocyte differentiation, lipid and carbohydrate metabolism, and adipokine synthesis, including three isoforms encoded by different genes (Eldor et al., 2013). PPARα is highly expressed in liver, kidney, and skeletal muscle. PPARγ is also highly expressed in various cell types and organs, including fat cells, muscle cells, liver, and kidneys, and is considered to be a major regulator of glucose homeostasis (Haluzík and Haluzík, 2006). Studies have suggested that the production of future PPARα and γ double agonists will simultaneously bring about favorable changes in PPARα lipid mass spectrometry and blood glucose benefits of PPARγ agonists (Massaro et al., 2016). The existing insulin-sensitizing drug thiazolidinedione is a potent agonist of nuclear PPAR-γ (Hevener et al., 2003). Based on the traditional anti-diabetic formula, TZQ-F can improve IR in KKA(y) mice through its efficacy in regulating adipocyte differentiation and insulin action, and the results indicate that its therapeutic effect is related to the upregulation of PPARγ expression in liver tissue (Nan Xia et al., 2013) and PPARγ coactivator 1α (PGC1α), which is a PPAR-mediated transcriptional coactivator of fatty acid oxidation (Koo et al., 2004). Tang-Nai-Kang is a mixture of five herbal plant extracts that has been shown to improve glucose metabolism abnormalities in patients with pre-diabetes. Tang-Nai-Kang treatment can deacetylate PGC1α to activate it and synergize with PPAR expression to enhance fatty acid oxidation and improve insulin levels in rats (Li et al., 2015).

## Conclusions and Perspectives

IR is a pathological condition common to many metabolic diseases; the most well known of which is T2D. Some surveys show that China is one of the countries with the highest incidence of T2D in the world, and the number of people with diabetes in the adult population have exceeded 113.9 million in 2010 (Zheng et al., 2018). The global trend of other metabolic diseases associated with IR is also not optimistic. From 2003 to 2012, the overall prevalence of metabolic syndrome in the United States was 33% (Aguilar et al., 2015). It is estimated that nearly 100 million people in the United States have NAFLD (Rinella, 2015). Global Burden of Disease studies have shown that the prevalence of obesity has doubled in 73 countries between 1980 and 2015 (Inoue et al., 2018). In addition, based on the 1990 National Institutes of Health standard, PCOS affects 6–10% of women worldwide (Goodarzi et al., 2011), and studies have indicated that 56.3% of Han women in China with PCOS have IR (Li et al., 2018). Therefore, in the face of such a large potential patient population, the harm caused by IR cannot be ignored. Herbs have been used in China for more than 2,000 years and are still considered effective drugs to prevent and treat various diseases. The discovery and application of artemisinin, a compound derived from *Artemisia annua*, is a good example. In recent years, research on Chinese medicine has gradually been standardized and systematized. China’s latest guidelines for preventing and treating T2D are also the first to include Chinese medicine treatment.

The information presented in this review shows that herbal formulas, active ingredients, and natural products can be effective to improve IR. Targets of herbal compounds that affect insulin signaling include insulin receptor substrate, phosphatidylinositol 3-kinase, glucose transporter, AMPK, glycogen synthase kinase 3, MAPKs, JNK, NF-κB, protein tyrosine phosphatase 1B, nuclear factor-E2-related factor 2, and peroxisome proliferator-activated receptors.

In **Table 7**, we have listed the composition of the above herbal formula in detail. In addition, we have found that certain herbs have a good application prospect in the treatment of IR. Such single botanicals include Coptis, Pueraria, Mulberry, Salvia, and others. Effective extracts of herbal medicines include berberine and mulberry leaves. There are also effective active ingredients such as berberine, ginsenoside, astragaloside, and resveratrol. The effects of these drugs are not only limited to enhancing insulin sensitivity, but also can be beneficial for improving systemic metabolism, such as reducing fasting blood glucose and postprandial blood glucose, improving blood lipid metabolism, reducing body weight, lowering blood pressure, and regulating female hormone secretion. Therefore, the role of herbal medicine in the treatment of IR is not only beneficial for treating T2D, but also provides new ideas for treating obesity, metabolic syndrome, PCOS, and NAFLD.

**Table 7:** Composition of herbal formula.

JTTZ formula	*Aloe vera*, *Coptis chinensis*, *Rhizoma Anemarrhenae*, red yeast rice, *Momordica charantia*, *Salvia miltiorrhiza*, *Schisandra chinensis*, *Zingiber oj-jicinale Rosc.*
Tangyiping granules (TYP)	*Astragalus mongholicus* 30 g, *Paeoniae Radix Alba* 12 g, *Coptis chinensis* 15 *g*, *Salvia miltiorrhiza* 12 g, *Pinellia ternate* 9 *g*, *Pueraria lobata* 30 g.
Jinlida (JLD)	*Panax ginseng C. A. Mey.*, *puerarin*, *pale white atractylodes rhizome*, *Coptis chinensis*, *poria cocos*, *radix polygonati officinalis*, and so on
Qingxue Dan (QXD)	*Scutellaria baicalensis Georgi*, *Coptis chinensis Franch.*, *Platycladus orientalis (Linn). Franco*, *Gardenia jasminoides Ellis*, *Rhizoma of Rheum palmatum Linne*
Qingre Yangyin Recipe (QRYYR)	*Cortex Lycii*, *Rehmannia glutinosa Libosch*, *Ophiopogon japonicus (Linn. f). Ker-Gawl.*,*Cynanchum otophyllum*, *Salvia miltiorrhiza*,*Poria cocos(Schw).Wolf*, *Acorus tatarinowii*, *Alisma plantago-aquatica Linn.*, *Lycium barbarum L.*,*Cuscuta chinensis Lam.*,*Epimedium sagittatum*, *Rubus idaeus L.*
Sancaijiangtang powders	*Panax Ginseng*,*Asparagus Racemosus*, *Radix*, *Rehmanniae*,**dark plum fruit, *Cortex*, *Cinnamomi*, *Rhizoma Coptidis*,
Jinlida	*Panax ginseng C. A. Mey.*, *Fallopia multiflora (Thunb). Harald*, *Atractylodes lancea (Thunb). DC*, *Sophora flavescens*, *Ophiopogon japonicus (Linn. f). Ker-Gawl.*, *Rehmannia glutinosa Libosch*, *Fallopia multiflora (Thunb). Harald*, *Cornus officinalis Sieb. et Zucc.*, *Poria cocos (Schw). Wolf*, *Eupatorium fortunei Turcz.*,*Coptis chinensis Franch.*, *Anemarrhena asphodeloides Bunge*, *Epimedii Folium*, *Salvia miltiorrhiza Bge.*, *Pueraria thomsonii Benth*, *Litchi chinensis Sonn.*, *Cortex Lycii*
Zhenggan Tang decoction	*Pseudostellaria heterophylla (Miq). Pax ex Pax et Hoffm.*, *Atractylodes macrocephala Koidz.*, *Astragalus mongholicus Bunge*, *Poria cocos (Schw). Wolf*, *Angelica sinensis (Oliv). Diels*, *Salvia miltiorrhiza Bge.*, *Trionyx sinensis Wiegmann*, *Radix Bupleuri*, *Cynanchum otophyllum*
xin-ju-xiao-gao-fang (XJXGF)	*Rheum palmatum L.*, *Coptis chinensis Franch.*,*Cassia tora Linn.*, *Citrus aurantium L.*
Yiqi Huaju Recipe (YHR)	*Astragali Radix 10 g*, *Coptis chinensis Franch.* 3* g*, *Typha angustifolia L.* 10* g*, *Artemisia capillaris Thunb* 10 g, *Alisma plantago-aquatica Linn.* 10 g
Yangxin Tongmai Formula (YTF)	*Radix Ginseng* 10* g*, *Radix Salviae Miltiorrhizae* 15* g*, *Ramulus Cinnamomi* 6* g*, *Fructus Aurantii Immaturus* 10* g*, *Rhizoma Alismatis* 10 g
Sancai powder	*Radix Ginseng*, *Radix Asparagi*, *Cochinchinensis*, *Radix Rehmanniae*, *Rhizoma Coptidis*, *Cortex Cinnamomi Cassiae*, *Fructus Mume*
SGY preparation	*Morus alba L.*, *Pueraria thomsonii Benth (Leguminous)*, *Dioscoreae rhizoma (Dioscoreaceae)*, *Momordica charantia L.*
Dai-Zong-Fang	*Rhizoma Coptidis*, *Fructus Aurantii Immaturus*
Zengye Decoction (ZYD)	*Radix Scrophulariae*, *Radix Rehmanniae*, *Radix Ophiopogonis*
Jia-Wei-Jiao-Tai-Wan (JWJTW)	*Cinnamomum cassia*, *Rhizoma coptidis*, *Astragalus membranaceus*, *Herba Gynostemmatis*, *Radix Puerariae Lobatae*, *Folium Mori*, *Semen Trigonellae*
Seyoeum (SYE)	*Coix lacryma-jobi*, *Oryza sativa*, *Sesamum indicum*, *Glycine max*, *Liriope platyphylla*, *Dioscorea batatas*
Wu-Mei-Wan	*Lycium barbarumL.*, *Angelica dahurica (Fisch. ex Hoffm.)Benth. et Hook. f. ex Franch. et Sav*, *Zingiber oj-jicinale Rosc.*,*Rhizoma coptidis*, *Angelica sinensis (Oliv). Diels*, *Ziziphus jujuba Mill.*, *Zanthoxylum bungeanum Maxim.*,*Cinnamomum cassia*, *Panax ginseng C. A. Mey.*, *Platycladus orientalis (Linn). Franco*
Preparation JQ-R	*Rhizoma Coptidis*, *Astragalus membranaceus*, *Lonicera japonica*
Fenugreek seed and mulberry leaf	*Morus alba L.*, *Trigonella foenum-graecum L.*, *Panax quinquefolius L.*
Erchen Decoction and Linguizhugan Decoction	*Pinellia ternata*, *Pericarpium Citri Reticulatae*, *Poria cocos (Schw). Wolf*, *Glycyrrhiza uralensis Fisch.*/*Poria cocos*, *cassia twig*, *Rhizoma Atractylodis Macrocephalae*, *and licorice*
Jiao-Tai-Wan (JTW)	*Rhizome Coptidis*, *Cortex Cinnamomi*
Modified lingguizhugan decoction	*Poria cocos (Schw). Wolf*, *Cinnamomum cassia Presl*, *Atractylodes lancea (Thunb). DC.*, *Glycyrrhiza uralensis Fisch.*, *Codonopsis pilosula (Franch). Nannf.*, *d Rheum palmatum L*
Jiang Tang Xiao Ke (JTXK) granule	*Radix rehmanniae*, *Fructuscorni*, *Radix salviae miltiorrhizae*, *Rhizoma coptidis*, *Radix Puerariae Lobatae*, *etc.*
Jiangzhi Capsule	*Radix Astragali*, *Poria cocos (Schw). Wolf*, *Folium Nelumbinis*, *Rhizoma Alisma*, *Fructus Crataeg*, *Fructus Chaenomelis*, *Radix et Rhizoma Salviae Miltiorrhizae*, *Radix et Rhizoma Notoginseng*, *Pollen Typhae*, *Rhizoma et Radix Polygoni cuspidate*, *Herba Taraxaci*, *Radix Polygoni multiflori*, *Fructus Ligustri Lucidi*
Liuwei Dihuang decoction	*Rehmannia glutinosa Libosch*, *Cornus officinalis Sieb*, *Dioscorea opposite Thunb*, *Alisma orientale Juz*, *Poria cocos Wolf*, *Paeonia suffruticosa Andrews*
ZiBu PiYin Recipe (ZBPYR)	Red Ginseng, *Common Yam Rhizome*, *Poria cocos (Schw). Wolf*, *Cynanchum otophyllum*, *Salvia miltiorrhiza Bge.*, *Dolicho Lablab L.*, *Nelumbo nucifera Gaertn.*, *Acorus tatarinowii*, *Polygala tenuifolia Willd.*, *Santalum album linn*, *Pericarpium Citri Reticulatae*, *Glycyrrhiza uralensis Fisch.*
Jinlida	*Panax ginseng C. A. Mey.*, *puerarin*, *pale white atractylodes rhizome*, *Coptis chinensis*, *poria cocos*, *radix polygonati officinalis*, *etc.*
Shouwu Jiangqi Decoction (SJD)	*Fallopia multiflora (Thunb).*, *Harald*, *Astragalus membranaceus (Fisch).*, *Bunge.*, *roasted Bombyx Batryticatus*, *Common Yam Rhizome*, *Euonymus alatus (Thunb). Sieb.*, *Cyperus rotundus L.*, *etc.*
Tang-Nai-Kang (TNK)	*Fructus Ligustri Lucidi*, *Prunella vulgaris L.*, *Saururus chinensis (Lour). Baill*, *Psidium guajava Linn.*, *Panax ginseng C. A. Mey.*
Jinlida (JLD)	*Panax ginseng C. A. Mey.*, *Polygonatum sibiricum*, *Atractylodes Lancea (Thunb). DC.*, *Sophora flavescens*, *Ophiopogon japonicus (Linn. f). Ker-Gawl.*, *Rehmannia glutinosa Libosch*, *Fallopia multiflora (Thunb). Harald*, *Cornus officinalis Sieb. et Zucc.*, *Poria cocos (Schw). Wolf.*, *Eupatorium fortunei Turcz.*, *Coptis chinensis Franch.*, *Anemarrhena asphodeloides Bunge*, *Epimedium brevicornu Maxim.*, *Salvia miltiorrhiza Bge.*, *Pueraria thomsonii Benth*, *Litchi chinensis Sonn.*, *Cortex Lycii*
Modified Si-Miao-San (mSMS)	*Coptis chinensis Franch.*, *Phellodendron amurense Rupr.*, *Semen Coicis*, *Atractylodes Lancea (Thunb). DC.*
Gal-geun-dang-gwi-tang (GGDGT)	*Pueraria thomsonii Benth*, *Glycyrrhiza uralensis Fisch.*, *Angelica sinensis (Oliv). Diels*, *Ophiopogon japonicus (Linn. f). Ker-Gawl.*, *Cynanchum otophyllum*, *Cornus officinalis Sieb. et Zucc.*, *Chaenomeles sinensis (Thouin) Koehne*, *Rehmannia glutinosa Libosch*, *Nelumbo nucifera Gaertn*, *Dark Plum Fruit*, *Schisandra chinensis*, *Anemarrhena asphodeloides Bunge*, *Ligusticum chuanxiong Hort.*, *Asparagus cochinchinensis (Lour.) Merr.*, *Trichosanthes kirilowii Maxim*, *Cyperus rotundus L.*
Gyeongshingangjeehwan 18 (GGEx18)	*Laminaria japonica*, *Rheum palmatum L.*, *Ephedra sinica Stapf*
Salvia-Nelumbinis naturalis (SNN)	*Salvia miltiorrhiza Bge.*, *Nehlmbo nucifera*,*Reynoutria japonica Houtt.*, *Artemisia capillaris Thunb*
Kangen-karyu and Salviae Miltiorrhizae Radix	*Cynanchum otophyllum*, *Ligusticum chuanxiong Hort.*, *Carthamus tinctorius L.*, *Cyperus rotundus L. Radix Aucklandiae*, *Salvia miltiorrhiza Bge./Salvia miltiorrhiza Bge.*
Bushen Huatan Recipe (BHR)	*Epimedii Folium*, *Curculigo orchioides Gaertn*, *Atractylodes Lancea (Thunb). DC.*, *Pinellia ternata (Thunb). Breit.*,*Pericarpium Citri Reticulatae*, *Acorus calamus L.*, *Cyperus rotundus L.*, *Ligusticum chuanxiong Hort.*, *Alisma plantago-aquatica Linn.*, *Cervus nippon Temminc*, *Arisaema heterophyllum Blume*, *Amomum villosum Lour.*, *etc.*
Fu Fang Zhen Zhu Tiao Zhi formula (FTZ)	*Fructus Ligustri Lucidi*, *Atractylodes macrocephala Koidz.*, *Salvia miltiorrhiza Bge.*, *Coptis chinensis Franch.*, *Panax notoginseng (Burk). F.H. Chen*, *Eucommia ulmoides*, *Cirsium japonicum Fisch. ex DC*, *Citrus medica L.* var.* sarcodactylis Swingle*
Fructus Mume formula and its separated prescription	*Prunus mume (Sieb). Sieb. et Zucc.*, *Asarum heterotropoides*, *Zingiberis officinale Rosc*, *Coptidis chinensis Franch*, *Aconitum carmichaelii Debx.*, *Angelicas Sinensis (Oliv). Diels*, *Zanthoxylum bungeanum Maxim.*, *Cinnamomum cassia Presl*, *Panax ginseng C. A. Mey.*, *Phellodendrom chinense Schneid*
Refined-JQ (JQ-R)	*Rhizoma Coptidis*, *Astragalus membranaceus*, *Lonicera japonica*
TZQ-F	*Morus alba L.*, *Lotus Leaf*,*Salvia miltiorrhiza Bge.*, *Grataegus pinnati fida Bge.*, *Radix paeoniae rubra*
Jiaotai Pill (JTP)	*Coptis chinensis Franch.*, *Cinnamomum cassia Presl*
Heqi San	*Schisandra chinensis (Turcz). Baill.*, *Cynanchum otophyllum C. K. Schneid.*, *Hordeum vulgare L*
Fructus Mume formula	*Prunus mume (Sieb).Sieb. et Zucc.*, *Asarum heterotropoides Fr. Schmidt* var.* mandshuricum*, *Zingiberis officinale Rosc*, *Coptidis chinensis Franch*, *Aconitum carmichaelii Debx.*, *Angelicas Sinensis (Oliv). Diels*, *Zanthoxylum bungeanum Maxim.*, *Cinnamomum cassia Presl*, *Panax ginseng C. A. Mey.*, *Phellodendrom chinense Schneid*

However, there were some shortcomings in our research. First, most of the interventions studied were herbal formulas and extracts. The diversity of ingredients in the herbal formulas and extracts results in complex potential therapeutic mechanisms. Not only does it make understanding the role of drugs difficult, but it also makes in-depth research impossible. However, the existing research also has the same shortcomings as our research. In addition, there are studies questioning the role of herbal medicine in the treatment of IR. The beneficial effects of ginsenosides on IR are listed in **Table 4**, but clinical trials have shown that oral ginseng or ginsenosides do not improve insulin sensitivity in glucose-tolerant or obese/overweight subjects who are newly diagnosed with diabetes (Reeds et al., 2011). However, the clinical trial lasted only 8 weeks, and in most *in vitro* and *in vivo* tests, ginsenosides were administered by intraperitoneal injection. In clinical trials, the systemic utilization and metabolic processes have to be considered only after oral administration. Studies have indicated that ginsenoside is a precursor. The pharmacological action of ginsenoside is to activate intestinal deglycosylation and fatty acid esterification (Hasegawa, 2004). Therefore, the *in vitro* and *in vivo* levels of ginsenosides should not be the only focus of the trial.

In response to these shortcomings, we can make some improvements in future studies. For example, regarding the bioavailability of herbal medicines, we can use targeted drug delivery systems (TDDSs) to improve the way in which the active ingredients of herbal medicines are administered. Research on targeted preparations related to TDDSs has become a popular topic, especially for applications in the field of anti-cancer research. Targeted administration can be divided into liposomes, granules, nanoparticles, emulsions, and other similar preparations (Li et al., 2009). The combination of drugs and specific target carriers can play a synergistic and attenuating role. At present, research on herbal drug delivery systems is still in the exploration stage, and the design, synthesis, and quality evaluation of TDDS are more suitable for single herbal ingredients. Therefore, separating the active ingredients of herbs is particularly important, including those known or unknown. We can select safe and effective single herbs from the many herbal formulas obtained from the literature and experimental research and separate the active ingredients, such as monomers, by pharmacological methods. Then, based on the active ingredients of the herbal medicine, the effective target mechanism of the drug can be studied. Finally, the active ingredient, target, and TDDS are combined. This not only makes the research on herbal medicine more targeted and efficient, but also provides a good prospect for the development and application of anti-insulin drugs.

## Author Contributions

JL and LB designed the work of review. JL, LB, and FW reviewed the literature available on this topic and wrote the paper. JZ, DW, YX, and WY contributed in the scientific writing of the manuscript. JL, LB, and JW revised the manuscript. All authors approved the paper for publication. JL, LB, FW, JZ, DW, YX, and WY contributed equally to this work. JL and LB contributed equally to this study and share first authorship.

## Funding

This paper was supported by Central Health Research Project W2017BJ43.

## Conflict of Interest Statement

The authors declare that the research was conducted in the absence of any commercial or financial relationships that could be construed as a potential conflict of interest.
